# Antioxidant and Laxative Effects of Methanol Extracts of Green Pine Cones (*Pinus densiflora*) in Sprague-Dawley Rats with Loperamide-Induced Constipation

**DOI:** 10.3390/antiox14010037

**Published:** 2024-12-31

**Authors:** Hee-Jin Song, Ayun Seol, Jumin Park, Ji-Eun Kim, Tae-Ryeol Kim, Ki-Ho Park, Eun-Seo Park, Su-Jeong Lim, Su-Ha Wang, Ji-Eun Sung, Youngwoo Choi, Heeseob Lee, Dae-Youn Hwang

**Affiliations:** 1 Department of Biomaterials Science (BK 21 FOUR Program), Life and Industry Convergence Research Institute, College of Natural Resources and Life Science, Pusan National University, Miryang 50463, Republic of Korea; hejin1544@naver.com (H.-J.S.); a990609@naver.com (A.S.); prettyjiunx@naver.com (J.-E.K.); xofuf0701@naver.com (T.-R.K.); pujihao@naver.com (K.-H.P.); geg9393@naver.com (E.-S.P.); soojl1315@naver.com (S.-J.L.); dhkdtngk@naver.com (S.-H.W.); ssungji@pusan.ac.kr (J.-E.S.); ychoi@pusan.ac.kr (Y.C.); 2 Department of Food Science and Nutrition, College of Human Ecology, Pusan National University, Busan 46241, Republic of Korea; mminv8@naver.com (J.P.); heeseoblee@pusan.ac.kr (H.L.)

**Keywords:** pine cones, diterpenoids, antioxidants, constipation, laxative effects, stools

## Abstract

Oxidative stress is the key cause of the etiopathogenesis of several diseases associated with constipation. This study examined whether the green pine cone can improve the symptoms of constipation based on the antioxidant activities. The changes in the key parameters for the antioxidant activity and laxative effects were examined in the loperamide (Lop)-induced constipation of Sprague-Dawley (SD) rats after being treated with the methanol extracts of green pine cone (MPC, unripe fruits of *Pinus densiflora*). MPC contained several bioactive compounds, including diterpenoid compounds such as dehydroabietic acid, taxodone, and ferruginol. In addition, it exhibited high scavenging activity against 2,2-diphenyl-1-picrylhydrazyl (DPPH) and 2,2′-azino-bis (3-ethylbenzothiazoline-6-sulfonic acid) (ABTS) radicals. These effects of MPC successfully reflected the improvement in nicotinamide adenine dinucleotide phosphate oxidase (NADP) H oxidase transcription, superoxide dismutase (SOD) levels, and nuclear factor erythroid 2-related factor 2 (Nrf2) phosphorylation levels in the mid colon of Lop+MPC-treated SD rats. Furthermore, significant improvements in the stool parameters, gastrointestinal (GI) transit, intestine length, and histopathological structure of the mid colon were detected in the Lop-induced constipation rats after MPC treatment. The other parameters, including the regulators for the adherens junction (AJ) and tight junction (TJ), and GI hormone secretion for laxative effects, were improved significantly in Lop+MPC-treated SD rats. These effects were also verified in Lop+MPC-treated primary rat intestine smooth muscle cells (pRISMCs) through analyses for antioxidant defense mechanisms. Overall, the finding of this study offers novel scientific evidence that MPC could be considered as a significant laxative for chronic constipation based on its antioxidant activity.

## 1. Introduction

Oxidative stress can be defined as the overproduction of reactive oxygen species (ROS, free radicals) because of an imbalance between the production of oxidants and the antioxidant defense mechanism [1,2]. During these processes, ROS and other oxidants can induce the peroxidation of DNA, proteins, and lipids within various components and organelles of cells, followed by membrane damage and cell death, leading to tissue damage [3,4]. Because of these properties of ROS, this stress is considered one of the major causes of chronic diseases, including cardiovascular disease, diabetes, and neurodegenerative disorders, through the enhancement of biomarkers for oxidative damages and risk factors for the development of pathological conditions [5]. Among these diseases, gastrointestinal (GI) dysmotility-related diseases, including constipation, have attracted considerable attention because the colon is susceptible to damage induced by oxidative stress [6]. The prominent symptoms of chronic constipation and its associated diseases are considered warning signals induced by oxidative stress [7,8]. The phenotypes of constipation were remarkably attenuated in the constipation model with oxidative stress after treatment with prebiotics [9]. In addition, significant improvements in oxidative stress and growth retardation of children with constipation were improved by advanced medication for a prolonged duration [10,11]. Under persisting oxidative stress, multiple cell types, such as enteric neurons, muscle cells, and epithelial cells in the GI tract, suffer from various levels of damage [12]. This stress can cause intrinsic nitrosative injury, mitochondrial dysfunction, or inflammation-related pathways in the enteric nervous system, while significant alterations in muscle relaxation and contraction, atrophy, and cell death were detected in the gastric smooth muscle cells [13,14,15]. Furthermore, oxidative stress can lead to remarkable damage, including tight junction injuries and systemic endogenous stress syndrome in intestinal epithelial cells [16].

Several compounds and natural products with high antioxidant activity have been evaluated for their potential as constipation treatments based on scientific evidence for the correlation between constipation and oxidative stress. Among them, most natural products, including *Malva sylvestris*, Ramie leaf (*Boehmeria nivea* L.), *Ecklonia cava*, and *Cistanche deserticola*, exhibited the promoting effects on these linkages [17,18,19,20]. Significant improvements in distinct constipation symptoms were detected, such as a decline in stool parameters, inhibition of GI transit, disruption of histopathological structure, suppression of mucin secretion, and decrease in the GI hormone, even though there are differences in the factors analyzed. At the same time, they enhanced antioxidant capacities, including an increase in the antioxidant enzyme activity, suppression of lipid peroxidation, and inhibition of H_2_O_2_ overload in the colon of animals [17,18,19,20]. Similar effects were detected in a few compounds derived from natural products. Taurine–xylose (T–X) and pterosilbene (PTE) improved the stool parameters and GI transit ratio and increased the antioxidant enzyme activity and transcriptional regulatory pathway in the loperamide (Lop)-induced constipation model [21,22]. On the other hand, despite several studies on the antioxidant, antiaging, anti-inflammatory, and antiviral activity as well as compositions of green pine cones (*Pinus densiflora*), there are no reports on whether green pine cones can induce antioxidant defense mechanisms and laxative effects simultaneously in a Lop-induced constipation model [23,24,25,26].

This study examined whether the methanol extracts of green pine cones (MPC) have antioxidative and laxative effects in a Lop-induced constipation model. Subsequently, the function of this product was further verified in the primary smooth muscle of rat intestine cells (pRISMC) after the Lop and MPC treatment.

## 2. Materials and Methods

### 2.1. Preparation and Extraction of MPC

Green pine cones, which were still greenish and not fully ripened, were collected from Mucheok Mountain in Gimhae, Korea, between July and August. Prof. Young-Whan Choi, Department of Horticultural Bioscience, Pusan National University (Miryang, Republic of Korea), confirmed the cone samples to be *Pinus densiflora*. MPC were extracted with a similar method as described in a previous study except for the ratio of solvent to powder [27]. The cones were halved, freeze-dried, and ground into a pine powder. The powder (15 g) was gently mixed with 300 mL of 80% methanol (*v*/*v*) and shaken overnight at 37 °C and 200 rpm in a shaking incubator (VS-8480; Vision Scientific, Bucheon, Republic of Korea). The supernatants of mixtures were filtered and concentrated through No. 2 filter paper (Whatman, Brentford, UK) and using a rotatory vacuum evaporator (N-1100 series; EYELA, Tokyo, Japan). Finally, the powder form of MPC was freeze-dried and stored at −20 °C until further experiments. Voucher specimens of *Pinus densiflora* (WPC-24-003) were deposited in the Functional Materials Bank of the Wellbeing RIS Center (FMB-WRIS Center) at Pusan National University.

### 2.2. Determination of Phytochemical Contents in MPC

The total phenolic content (TPC) of MPC was analyzed using the Folin–Ciocalteu method as described in a previous study [28]. MPC solution (1 mL) was combined with Folin–Ciocalteu reagent (5 mL; Sigma-Aldrich Co., St. Louis, MO, USA), and subsequently, 15 mL 20% Na_2_CO_3_ was added. The absorbance of the final solution was analyzed at 765 nm using a VersaMax plate reader (SoftMax^®^ Pro Software (ver. 5.2); Molecular Devices, Sunnyvale, CA, USA). And then, TPC in MPC were calculated based on the calibration curve of standard gallic acid (Sigma-Aldrich Co.).

In addition, the total flavonoid contents (TFC) in MPC were analyzed as described in a previous study [29]. The various concentrations of MPC solution (20 μL) were sequentially mixed with 5% NaNO_2_ (60 μL) and 10% AlCl_3_ (60 μL) (Sigma-Aldrich Co.). The absorbance of the final solution was detected with a VersaMax plate reader (Molecular Devices, Sunnyvale, CA, USA). And then, TFC in MPC were calculated using a standard calibration curve of catechin (Sigma-Aldrich Co.).

### 2.3. Liquid Chromatography-Mass Sepctrometry (LC-MS) Analyses

Liquid chromatography (LC) was carried out using an ACQUITY UPLC BEH C18 (2.1 × 100 mm, 1.7 μm) column (Waters, Milford, MA, USA) following the procedures described in a previous study [27]. Mass spectrometric detection (MS) was conducted using an Agilent 1290 Infinity high-performance liquid chromatography (HPLC) system (Agilent Technologies, Waldbronn, Germany) coupled with a hybrid quadrupole time-of-flight (Q-TOF) mass spectrometer (TripleTOF 4600; AB Sciex Pte. Ltd., Framingham, MA, USA). The source parameters of LC-MS were as follows: 300 °C of gas temperature, 9 L/min of gas flow, 45 psig of nebulizer pressure, 350 °C of sheath gas temperature, 11 L/min of sheath gas flow and [M–H] ions of negative mode. The scan source parameters were 4000 V of VCap and 175 V of fragmentor voltage.

### 2.4. Free-Radical Scavenging Activity Analysis

The scavenging activity of MPC against 2,2-diphenyl-1-picrylhydrazyl (DPPH) radicals was determined as described in previous research [30]. Briefly, twelve different concentrations (1 to 500 μg/mL) of MPC were combined with 0.1 mM DPPH (Sigma-Aldrich Co.) or the same volume of a 95% ethanol solution (control). After incubation for 30 min, their absorbances were detected at 517 nm using a VersaMax plate reader (Molecular Devices).

In addition, the scavenging activity of MPC against 2,2-azino-bis (3-ethylbenzthiazoline-6-sulfonic acid) (ABTS) radicals was measured as described in previous research [31]. Briefly, various concentrations (1 to 500 μg/mL) of MPC were combined with an ABTS working solution (250 μL). After incubating for 4 min, their absorbances were measured at 734 nm with a UV-visible spectrophotometer (UV–vis, Thermo Fisher Scientific Inc., Waltham, MA, USA).

The scavenging activity of MPC against DPPH and ABTS was represented as the half-maximal inhibitory concentration (IC_50_). Also, this value is the MPC concentration (μg/mL) that exerted a 50% decrease in DPPH and ABTS activity.

### 2.5. Animal Care and Use

The animal experimental protocols for constipated Sprague-Dawley (SD) rats were approved by the Pusan National University-Institutional Animal Care and Use Committee (PNU-IACUC; Approval Number PNU-2024-0501). SD rats (eight-week-old, male, *n* = 35) were kindly provided from Samtako BioKorea Co. (Osan, Republic of Korea) and bred and handled at the Pusan National University-Laboratory Animal Resources Center, which is registered in the Korea Food and Drug Administration (KFDA; Accredited Unit Number: 000231) and the Association for Assessment and Accreditation of Laboratory Animal Care (AAALAC, Accredited Unit Number: 001525). Tap water and a chow diet (Samtako BioKorea Co.) were provided to them *ad libitum* throughout the experiment period. All SD rats were bred under a specific pathogen-free (SPF) facility with a strict photoperiod (12 h cycle of night and day), temperature (23 ± 2 °C), and humidity (50 ± 10%).

### 2.6. Experiment Design for Constipated SD Rat

The animal experimental design for the antioxidant and laxative effects of MPC is described elsewhere [19,32]. Male SD rats were selected to rule out the effects of sex differences because the estrogen hormones can affect the incidence of constipation in females [33]. Briefly, eight-week-old SD rats (male, 270 g, *n* = 35) were arranged into a non-constipation group (No group, *n* = 7) or a constipation group (Lop group, *n* = 28). SD rats were subcutaneously received by Lop (4 mg/kg body weight) in 0.5% Tween 20 saline solution, given twice daily (9 a.m. and 6 p.m.) for three days to induce constipation. After that, the Lop-injected group was assigned into one of four groups: Lop+Vehicle-treated group (*n* = 7), Lop+low concentration MPC (LMPC)-treated group (*n* = 7), Lop+medium concentration MPC (MMPC)-treated group (*n* = 7), and Lop+high concentration MPC (HMPC)-treated group (*n* = 7). These three groups were administered orally as a single dose of 100, 200, or 400 mg/kg body weight MPC. The optimal concentration of MPC was determined as described in previous studies evaluating the efficacy of other parts of pine trees and [23,25] as well as our preliminary data (Supplementary Figure S1), although there are no studies on efficacy using green pine cone. The same volume of 1× phosphate-buffered saline (PBS) was treated into the Lop+Vehicle-treated group under the same pattern. At 9 a.m. on the fifth day, total drinking water, food, stools and urine were collected from each rat using the metabolic cage (Daejong Instrument Industry Co., Ltd., Seoul, Republic of Korea) of each rat in the subset group, and GI transit was analyzed using a charcoal meal (Supplementary Figure S2A). Finally, all SD rats were euthanized by CO_2_ gas (a minimum purity of 99.0%) based on the international standard guideline. The complete death of the SD rats was confirmed by perfect respiratory and cardiac arrest, or dilated pupils and a fixed body. In addition, the GI tracts were harvested from the subset group of SD rats for GI transit analyses, and the mid colon was harvested for further analyses.

### 2.7. Measurement of Basic Physiological Factors and Feeding Parameters

The body weight was analyzed in each rat of all experimental groups using an electronic balance (Mettler Toledo, Greifensee, Switzerland). In addition, the food weight and water volume were analyzed at 9 a.m. on the fifth day using an electrical balance and a measuring cylinder. After then, their average value was calculated based on the measured results. Moreover, at 9 a.m. on the fifth day, urine was collected and measured twice per sample.

### 2.8. Analyses of the Stool Parameters

To harvest uncontaminated stools, all SD rats were individually maintained in metabolic cages (Daejong Instrument Industry Co., Ltd.) as reported in a previous study [34]. Briefly, total stools were collected from each rat at 9 a.m. for two days and then weighed and counted twice. In addition, the morphological features of stools were further analyzed using digital images (Canon, Middlesex, UK). The water content of stools was calculated based on fresh and dry weight of them as follows:

Water content of stools = [(A − B)/A] × 100

where the weight of fresh stools was defined as A, and the weight of dry stools after drying at 60 °C for 12 h was defined as B.

### 2.9. Analysis of GI Motility

The GI motility was measured using the charcoal transit ratio described elsewhere with some modifications [34]. After fasting for 18 h prior to the experiment, except for water ad libitum, all constipated SD rats were orally administrated charcoal meal (0.5 mL, 3% charcoal suspension in 0.5% methylcellulose solution) (Sigma-Aldrich Co.). The rats were euthanized using CO_2_ after 30 min of treatment, and the entire intestine from the stomach to the anus was collected. The charcoal transit ratio for GI motility was determined based on the length of related parameters as follows:

Charcoal transit ratio (%) = [(A − B)/A] × 100

where A was the length of total intestine, and B was the transit distance of charcoal meal.

### 2.10. Histopathological Analysis of the Mid Colon

Histopathological analysis was performed as described in a previous study [34]. The mid colons were initially fixed in 10% formalin solution for 48 h, followed by trimming and embedding in paraffin wax. The embedded tissues were sliced into 4 μm thick, and then these tissues were stained with hematoxylin and eosin solution (H&E; Sigma-Aldrich Co.) or Alcian blue solution (IHC WORLD, Woodstock, MD, USA). After mounting, the morphological features and colors of each tissue were observed using optical microscopy and analyzed with the Leica Application Suite (Leica Microsystems, Heerbrugg, Switzerland). H&E-stained slides were examined for 3 to 5 samples per group under magnification of 100× and 400×.

### 2.11. Quantitative Real-Time PCR (RT-qPCR) Analysis

RT-qPCR analyses were conducted to confirm the relative quantities for each gene. The total RNA molecules were isolated using RNAzol (Tet-Test Inc., Friendswood, TX, USA), and then the complementary DNA (cDNA) was synthesized with a mixture including oligo-deoxythymidine (dT) primer (Thermo Fisher Scientific Inc.), 2′-deoxynuclease-5′-triphosphate (dNTP), and reverse transcriptase (Superscript II, Thermo Fisher Scientific Inc.). The specific region of the target gene was amplified using a cDNA template and 2 × Power SYBR Green (TOYOBO Co., Osaka, Japan) and specific primers (Supplementary Table S1). During these processes, the fluorescence intensity was detected as per the manufacturer’s protocol.

### 2.12. Western Blot Analysis

Western blot analyses were conducted to protein expressions described as a previous study [35]. The total tissue proteins were collected by homogenizing the mid colon tissue (40 mg) and Pro-prep Protein Extraction Solution (iNtRON Biotechnology Inc., Seongnam, Korea). The total protein concentration was determined in tissue homogenate using a bicinchoninic acid protein assay (BCA) kit (Thermo Fisher Scientific Inc.). Also, the protein (30 µg) sample was run by 10% dodecyl sulfate-polyacrylamide gel electrophoresis (SDS-PAGE) for 1.5 h. The separated proteins were transferred onto nitrocellulose membranes at 40 V for 2 h. The target proteins on the membrane were bound with the specific primary antibodies (Supplementary Table S2) at 4 °C, and subsequently with goat anti-rabbit immunoglobulin G (IgG) conjugated with horseradish peroxidase (HRP) (TransGen Biotech Co., Ltd., Beijing, China) for 1 h at room temperature. The intensity level of each protein on the membrane was detected using a FluorChem^®^ FC2 Imaging system (Alpha Innotech Corporation, San Leandro, CA, USA).

### 2.13. Superoxide Dismutase (SOD) Activity Analysis

The SOD activity of each group was detected using a SOD assay kit (Dojindo Molecular Technologies Inc., Kumamoto, Japan) based on the manufacturer’s protocols and previous study [36]. The serum was diluted to 1/5, 1/5^2^, 1/5^3^, 1/5^4^, 1/5^5^, and 1/5^6^ with a dilution buffer. The diluted sample was added to 96-well plates, followed by the addition of 200 µL of a water-soluble tetrazolium salt-1 (WST-1) working solution. After adding enzyme solution (20 μL), the mixture was incubated at 37 °C for 20 min, and the absorbance of each mixture was detected with a spectrophotometer at 450 nm. The final activity of SOD was then calculated using the formula,

SOD activity = [(A _blank 1_ − A _blank 3_) − (A _sample_ − A _blank 2_)]/(A _blank 1_ − A _blank 3_) × 100

A _blank 1_, A _blank 2_, and A _blank 3_ represent the absorbance values of the respective blank wells, and A _sample_ is the absorbance of the sample well.

### 2.14. Preparation of pRISMCs and Treatment of MPC and Abietic Acid (AbA)

The pRISMCs were isolated using a slight modification of the method described elsewhere [35]. After collecting small intestines of infant rats (three days old), their luminal contents were flushed with Ca^2+^-free Hanks solution (5.02 mmol/L KCl, 135 mmol/L NaCl, 2.13 mmol/L MgCl_2_, 10 mmol/L glucose, and 1 mmol/L hydroxyethyl piperazine ethane sulfonic acid (HEPES), pH 7.4). Following the removal of the mucosal layer, these tissues (about 3–5 cm) were incubated with digestion solution [1 mg/mL collagenase (Worthington Biochemical, Lakewood, NJ, USA), 0.5 mg/mL trypsin inhibitor (Sigma-Aldrich Co.), and 1 mg/mL bovine serum albumin (Sigma-Aldrich Co.)] at 37 °C for 30 min. The pellet containing pRISMCs was collected with centrifugation at 1000 rpm for 5 min, and then the cells were cultured in 10% fetal bovine serum contained in Dulbecco’s Modified Eagle’s Medium (DMEM; Hyclone; GE Healthcare Life Sciences, Logan, UT, USA).

The antioxidant effects of MPC during constipation were confirmed in pRISMCs. Briefly, the wells with a density of 3 × 10^5^ cells were treated with 20 μM Lop for 12 h at 37 °C. After removing the culture medium, pRISMCs were incubated with 25, 50, and 100 μg/mL MPC or 10, 20, and 40 mM AbA for a further 12 h and used for 2′,7′-dihydrofluorescein diacetate (DCFH-DA) staining analysis or collected to measure the expression levels of specific proteins (Supplementary Figure S2B). AbA were prepared as described in previous studies as the highest single compounds contained within MPC [37].

### 2.15. DCFH-DA Staining and Nitric Oxide (NO) Concentration Analysis

The intracellular ROS levels in pRISMCs were determined with DCFH-DA (Sigma-Aldrich Co.) staining analysis. The Lop+MPC-treated cells were incubated with 10 µM DCFH-DA for 30 min at 37 °C. The stained cells were detected by a fluorescent microscope (Evos m5000; Thermo Fisher Scientific Inc.) at 400× magnification, and their number was measured in two different fields of view (67,500 mm^2^) in each well.

The nitrite levels were used to indicate NO production. Briefly, pRISMCs in each well were treated with Lop and MPC as described elsewhere, and the supernatants were collected from them. The culture supernatant (100 µL) was mixed with modified Griess reagent (100 µL, Invitrogen, CA, USA), and their absorbance was detected at 540 nm using a VersaMax plate reader (Molecular Devices).

### 2.16. Statistical Analysis

The statistical significance of each experimental group was determined with the one-way analysis of variance (ANOVA) or the unpaired two-sample t-test. After assessing the normality using the Shapiro–Wilk test in SPSS 27.0 (IBM Co., Armonk, NY, USA), Tukey multiple comparisons tests were also applied to support these two analyses. All values used in this study are represented as the means ± SD. Furthermore, a *p* value was considered to be significant only if it was <0.05.

## 3. Results

### 3.1. Detection of Bioactive Components in MPC

**Figure 1:**
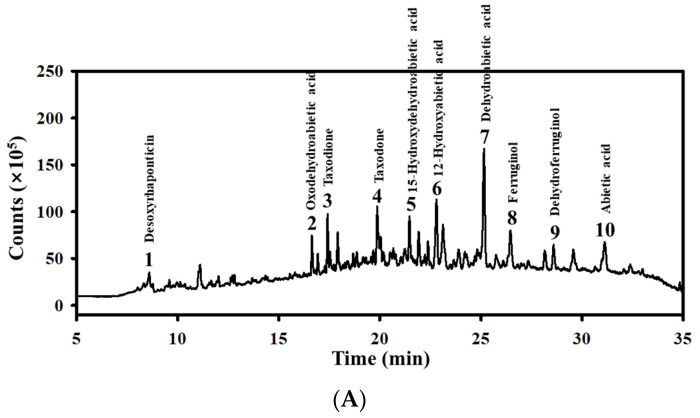

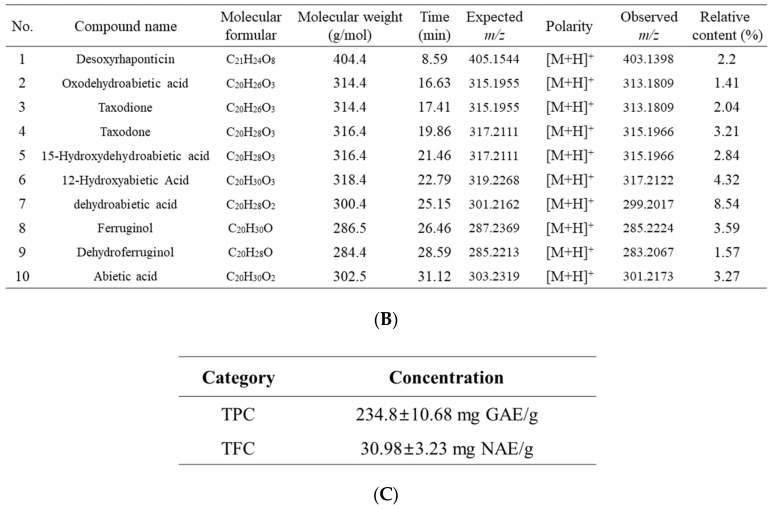
Detection of the bioactive components in the MPC. (**A**) LC-MS analysis of MPC. (**B**) Information of major bioactive components detected in MPC. (**C**) TPC and TFC in MPC.

First, the distributions of bioactive components in MPC were analyzed to predict its potential as an antioxidant for constipation. Approxi mately ten bioactive compounds were detected in MPC at different points using HPLC. Among them, dehydroabietic acid was detected as the compound with the highest amounts (8.56%), followed by 12-hydroxyabietic acid (4.32%), ferruginol (3.59%), abietic acid (3.27%), taxodone (3.21%), 15-hydroxydehydroabietic acid (2.84%), desoxyrhaponticin (2.2%), taxodione (2.04%), dehydroferruginol (1.57%), and oxodehydroabietic acid (1.41%) (Figure 1A,B) (Supplementary Figure S3). In addition, the TPC and TFC levels in MPC were 234.8 ± 10.68 mg GAE/g and 30.98 ± 3.23 mg NAE/g (Figure 1C). Therefore, the above results suggest that MPC has the potential for antioxidant activity and application as a therapeutic drug for constipation.

### 3.2. Radical Scavenging Activity of MPC

**Figure 2:**
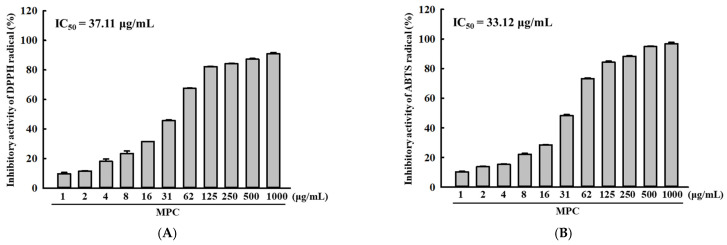
Scavenging activity of the MPC against free radicals. (**A**) DPPH and (**B**) ABTS radical scavenging activity. The activity of each radical was represented as IC_50_ value after determination the activity of the MPC (1–500 μg/mL).

The inhibitory activity against DPPH and ABTS radicals was measured in MPC to determine if the distribution of bioactive compounds is reflected in their antioxidant activity. These activities increased gradually at 1–500 μg/mL of MPC; the highest level was detected at 500 μg/mL. The IC_50_ value for DPPH radicals was determined to be 37.11 μg/mL of MPC, while the IC_50_ value for ABTS radicals was analyzed to be 33.12 μg/mL of MPC (Figure 2A,B). These results suggest that MPC has high antioxidative activity for free radicals.

### 3.3. Antioxidant Activities of MPC in the Mid Colon of Lop-Induced Constipation Rats

**Figure 3:**
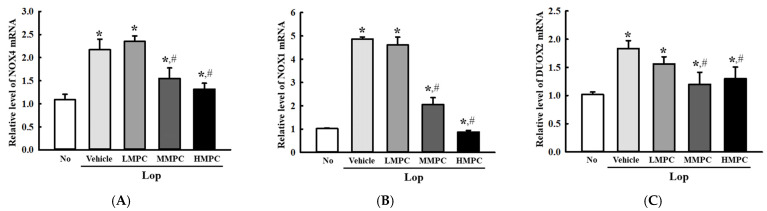
Transcription level of NAPDH oxidase genes in the mid colon of Lop+MPC-treated SD rats. (**A**) Transcription levels of *NOX4* gene. (**B**) Transcription levels of *NOX1* gene. (**C**) Transcription levels of *DUOX2* gene. These levels of three genes in the total mRNA were analyzed by RT-qPCR. The data are represented as the mean ± SD. * was represented a *p* value of less than 0.05 compared to the No group. # was represented a *p* value of less than 0.05 compared to the Lop+Vehicle-treated group.

**Figure 4:**
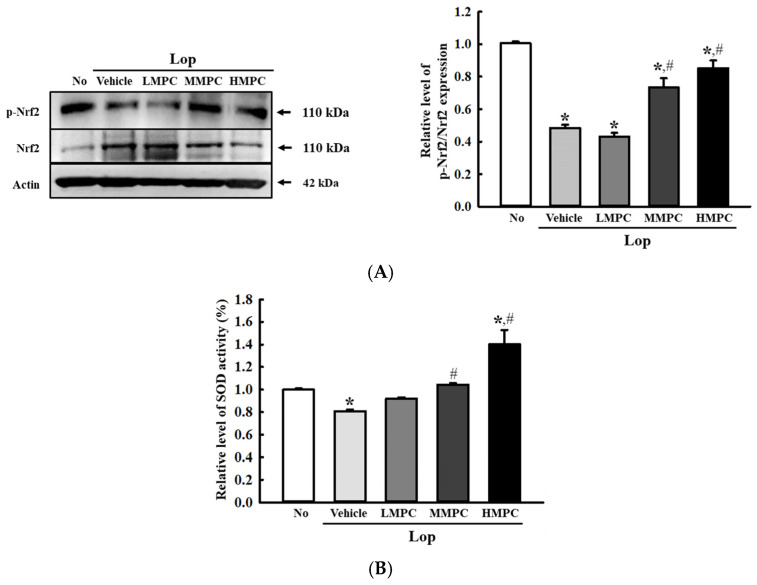
Expression of (**A**) p-Nrf2 and Nrf2 in the mid colon of Lop+MPC-treated SD rats. The expression level of each protein was measured in the mid colon by Western blot analysis using specific antibodies and represented as a relative value based on β-actin. (**B**) SOD activity in the serum of Lop+MPC-treated SD rats. SOD activity was measured in the serum of each rat. One unit of SOD activity is represented as the amount of the enzyme in the serum (20 µL). The data are represented as the mean ± SD. * was represented by a *p* value of less than 0.05 compared to the No group. # was represented by a *p* value of less than 0.05 compared to the Lop+Vehicle-treated group.

Next, we examined whether the radical scavenging activity and bioactive composition of MPC can help the activation of the antioxidant defense mechanism in the mid colon of constipated animals. To achieve this, the alterations in the levels of nicotinamide adenine dinucleotide phosphate (NADP) oxidase genes transcription, phosphorylation of transcription factor for the antioxidant enzymes, and SOD activity were analyzed in the mid colon of the Lop-induced constipation model after administration of MPC. The transcription levels of NADPH oxidase (NOX), *NOX1*, *NOX4*, and dual oxidase 2 (*DUOX2*) for NADP oxidase were higher in the Lop+Vehicle-treated group than in the No group. However, these levels were remarkably lower in the Lop+MPC-treated group than in the Lop+Vehicle-treated group (Figure 3A–C). In addition, a reverse regulation pattern was detected in nuclear factor erythroid 2-related factor 2 (Nrf2) phosphorylation and SOD activity. The decreased levels in the Lop+Vehicle-treated group were enhanced in the Lop+MPC-treated group, but the increase rate differed (Figure 4A,B). Therefore, the antioxidant activity of MPC can help activate the antioxidant defense mechanism in the mid colon of the constipated model.

### 3.4. Effects of MPC on the Feeding Behavior and Stool Parameters of Constipated SD Rats

**Figure 5:**
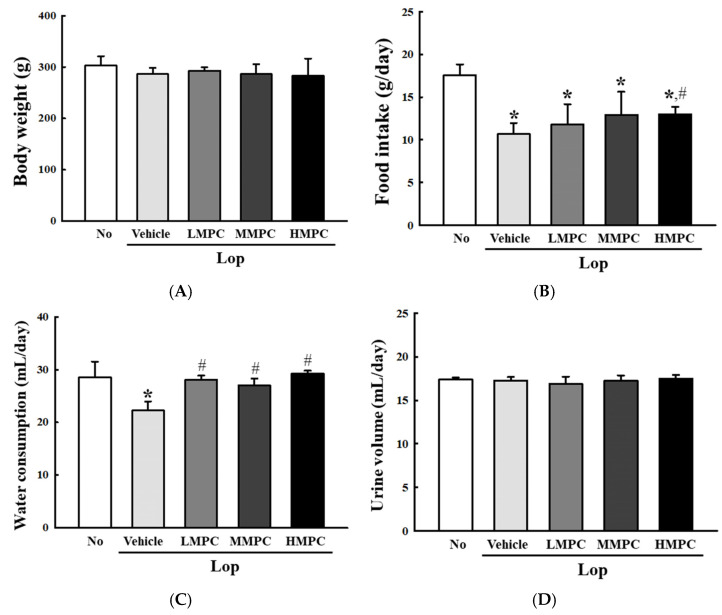
Body weight and feeding behavior of Lop+MPC-treated SD rats. (**A**) Body weight, (**B**) food intake, (**C**) water consumption, and (**D**) urine volume. The data are reported as the mean ± SD. * was represented by a *p* value of less than 0.05 compared to the No group. # was represented by a *p* value of less than 0.05 compared to the Lop+Vehicle-treated group.

**Figure 6:**
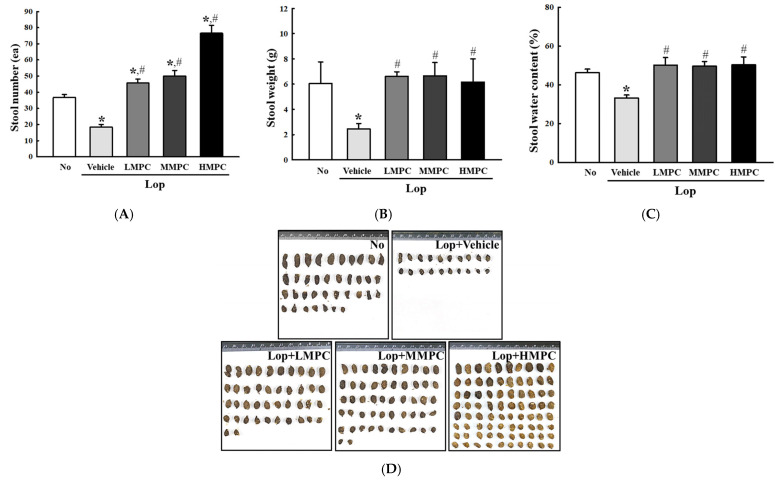
Stool parameters of Lop+MPC-treated SD rats. (**A**) Stool number. (**B**) Stool weight. (**C**) Stool water content. (**D**) Stool morphology. After collecting the stools from the metabolic cage, the digital images of stools were taken immediately, and their number and weight were analyzed using fresh stools. The data are represented as the mean ± SD. * was represented by a *p* value of less than 0.05 compared to the No group. # was represented by a *p* value of less than 0.05 compared to the Lop+Vehicle-treated group.

To investigate whether the administration of MPC with high antioxidant activity improves the excretion of constipated SD rats, alterations in feeding behavior and excretion parameters in Lop-induced constipation rats were analyzed after treatment with three different doses of MPC. The body weight and level of food intake were constantly maintained between the Lop+Vehicle-treated group and the Lop+MPC-treated group (Figure 5A,B). On the other hand, the amount of water consumption increased remarkably after MPC administration, even though the urine volume was maintained in all experimental groups (Figure 5C,D). In addition, some significant alterations were detected in stool parameters. Three key parameters, including weight, number, and water content for stools, were significantly lower in the Lop+Vehicle-treated group than in the No group. In contrast, MPC administration into constipated SD rats recovered these levels of three parameters, even though they did not exhibit dose-dependent changes (Figure 6A–C). The above changes in the stool parameters were reflected in the stool morphology. Small, round-shaped stools were transformed into long, oval-shaped stools after MPC administration (Figure 6D). Therefore, these results suggest that the administration of MPC with high antioxidant activity can improve the excretion of stools. These effects are also possible with very low MPC concentrations.

### 3.5. Effects of MPC on the GI Transit and Intestine Length of Constipated SD Rats

**Figure 7:**
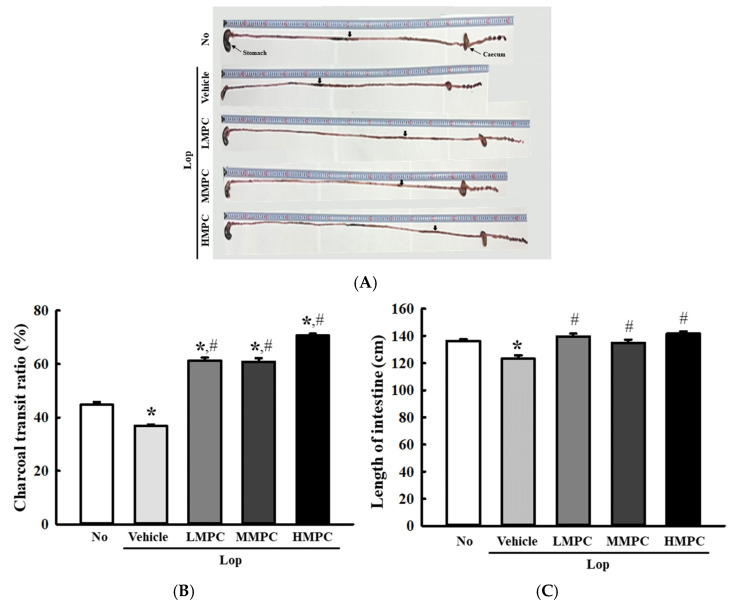
Phenotypes of GI in Lop+MPC-treated SD rats. (**A**) Digital image of GI tract showing the charcoal meal transit. After collecting total GI tract of SD rat treated with charcoal meal powder, their image was taken by a digital camera. The arrows indicate the transit location of the charcoal meal. (**B**) Transit ratio of the charcoal meal. This ratio was determined as described in materials and methods. (**C**) Length of the intestine. * was represented by a *p* value of less than 0.05 compared to the No group. # was represented by a *p* value of less than 0.05 compared to the Lop+Vehicle-treated group.

To examine if the defecation stimulation effects of MPC are accompanied by improving the GI function, the changes in GI transit ratio and intestinal length were measured in the Lop-induced constipation rats after treatment with three different MPC doses. These parameters were remarkably lower in the Lop+Vehicle-treated group than in the control. In contrast, the GI transit ratio and intestinal length were significantly increased in the three Lop+MPC-treated groups than in the Lop+Vehicle-treated group (Figure 7A–C). These results show that the defecation stimulation effects of MPC may be associated with improved GI motility and intestine length in Lop-induced constipated SD rats.

### 3.6. Effects of MPC on the Histopathological Structure of Mid Colon in Constipated SD Rats

**Figure 8:**
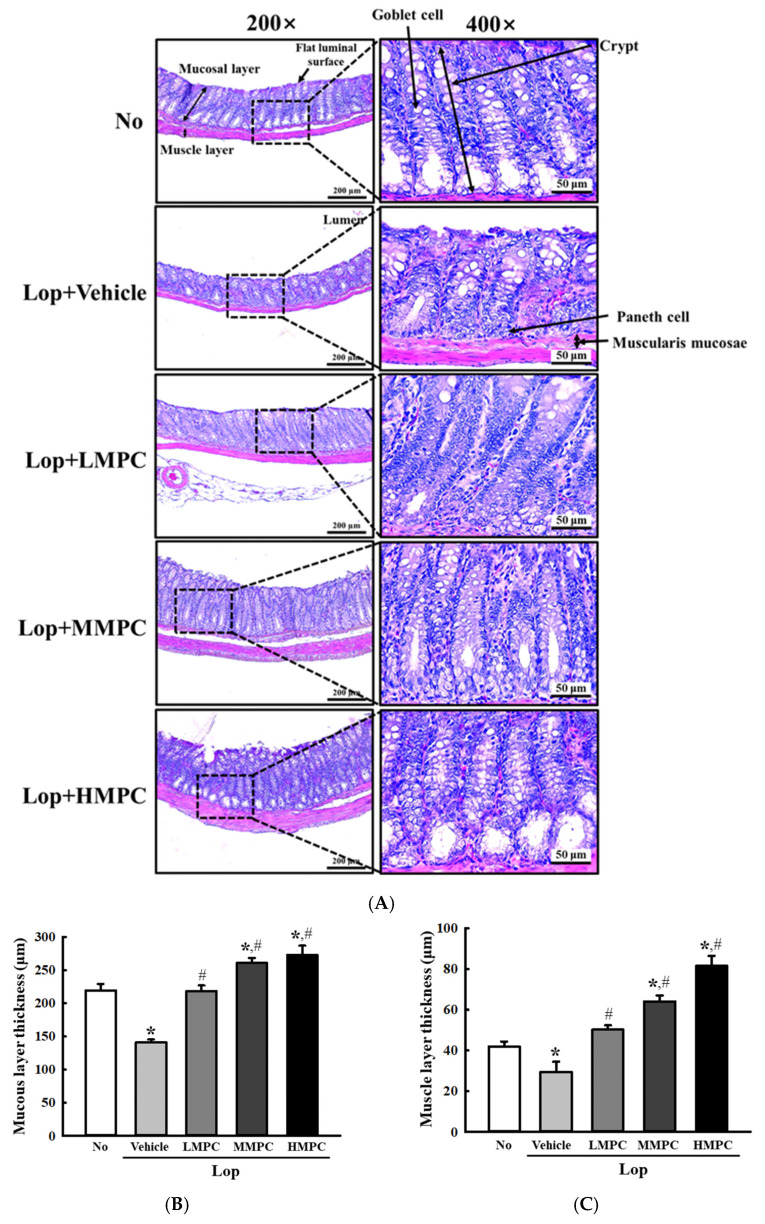

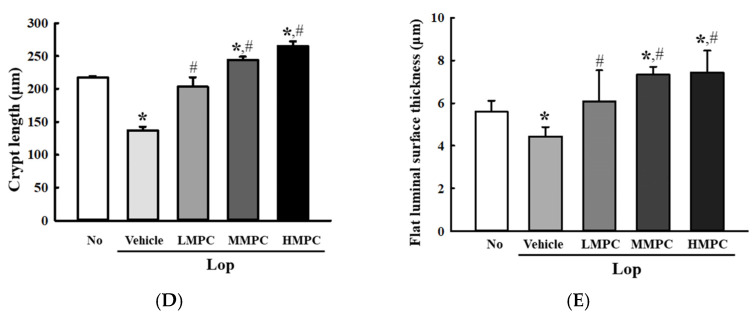
Histopathological structures of the mid colon in Lop+MPC-treated SD rats. (**A**) H&E-stained sections of the mid colon. The histological structure of the mid colon from each rat was observed using an optical microscope (100× and 400× magnification). (**B**–**E**) Histopathological parameters. The values of four parameters were analyzed using the Leica Application Suite. The data are represented as the mean ± SD. * was represented by a *p* value of less than 0.05 compared to the No group. # was represented by a *p* value of less than 0.05 compared to the Lop+Vehicle-treated group.

Next, we examined if the defecation stimulation effects of MPC are accompanied by alterations in the histopathological structure of the mid colon in the constipated model. To achieve this, alterations in the thickness of mucous, muscle, and flat luminal surface and crypt length were analyzed on the H&E-stained section of the mid colon of Lop-induced constipation rats after treatment with MPC. Significant alterations in the histopathological structure of the mid colon were observed on three parameters after MPC administration. Especially, remarkable changes were detected in the goblet cells and paneth cells. Some structural alterations, including a decrease in the cell number, morphological changes, and an irregular arrangement, were significantly recovered after MPC administration (Figure 8A). The decreased thicknesses of the mucous, flat luminal surface, and muscle in the Lop+Vehicle-treated group were enhanced in the Lop+MPC-treated group, even though there was a difference in the recovery rate (Figure 8B–E). Overall, these data suggest that the defecation stimulation effects of MPC may be linked to recovery on the histopathological structure of the mid colon of Lop-induced constipated SD rats.

### 3.7. Effects of MPC on the Mucin Secretion Ability and Water Retention Capacity in the Mid Colon of Constipated SD Rats

**Figure 9:**
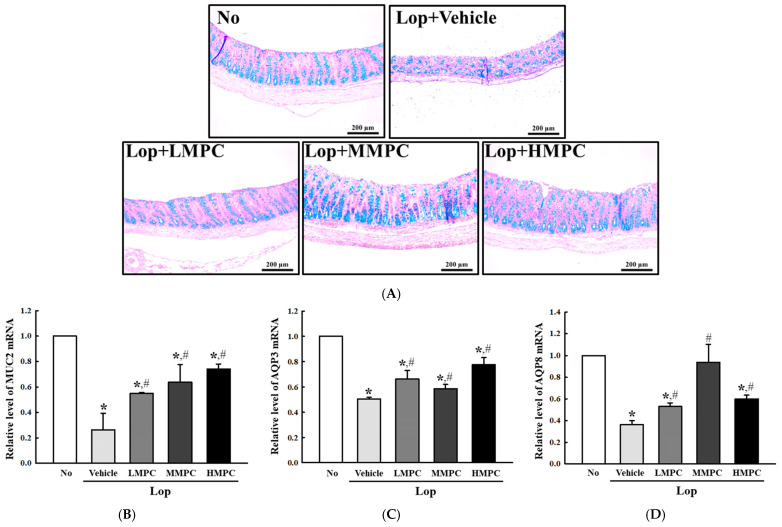
Mucin secretion and water capacity in the mid colon of the Lop+MPC-treated SD rats. (**A**) Alcian blue staining images. The levels of mucin were analyzed with staining of Alcian blue, and the blue stained images of mid colon were detected at 100× magnification. Alcian blue staining was prepared from mid colon of three to five rats per group, the density levels were observed in duplicate for each slide. (**B**) Transcription level of *MUC2*. (**C**) Transcription level of *AQP3*. (**D**) Transcription level of *AQP8*. These levels of three genes in the total mRNA were analyzed by RT-qPCR. The data are reported as the mean ± SD. * was represented by a *p* value of less than 0.05 compared to the No group. # was represented by a *p* value of less than 0.05 compared to the Lop+Vehicle-treated group.

To investigate whether defecation stimulation effects of MPC are accompanied by recoveries in the mucin secretion ability and water retention capacity in the mid colon of constipated models, alterations in the levels of the key parameters related to them were analyzed in the mid colon of Lop+MPC-treated SD rats. First, the dark blue color density for mucin in the mid colon was decreased in the Lop+Vehicle-treated group compared to the control group during the structural change in the mid colon. They were remarkably enhanced in the Lop+MPC-treated group when compared to the Lop+Vehicle-treated group (Figure 9A). In addition, the stained results with Alcian blue were reflected completely in the transcription levels of the mucin 2 (*MUC2*) gene. The decreased level of *MUC2* mRNA in the Lop+Vehicle-treated group increased significantly after MPC administration (Figure 9B). MPC also affected the water retention capacity of the mid colon. The transcription levels of the genes for aquaporin (*AQP*)*3* and *AQP8* were significantly enhanced in the three MPC-treated groups (Figure 9C,D). Therefore, the defecation stimulation effects of MPC may be associated with improved mucin secretion ability and water retention capacity of the mid colon of Lop-induced constipated SD rats.

### 3.8. Effects of MPC on the Structure and Function of Intestinal Epithelial Barrier in the Mid Colon of Constipated SD Rats

**Figure 10:**
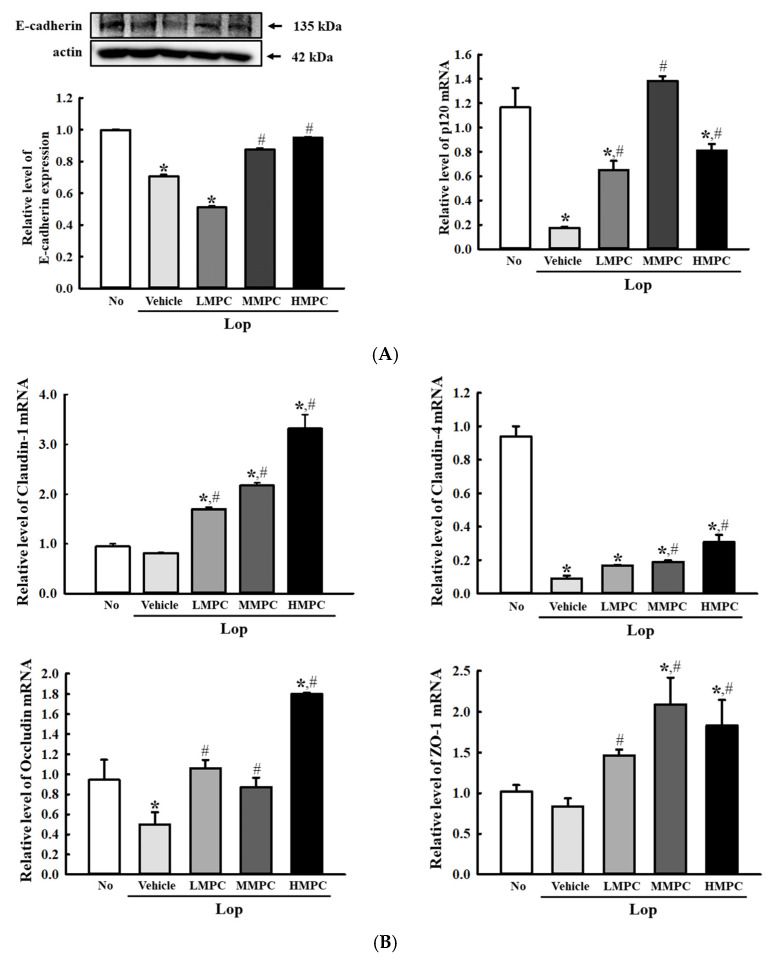

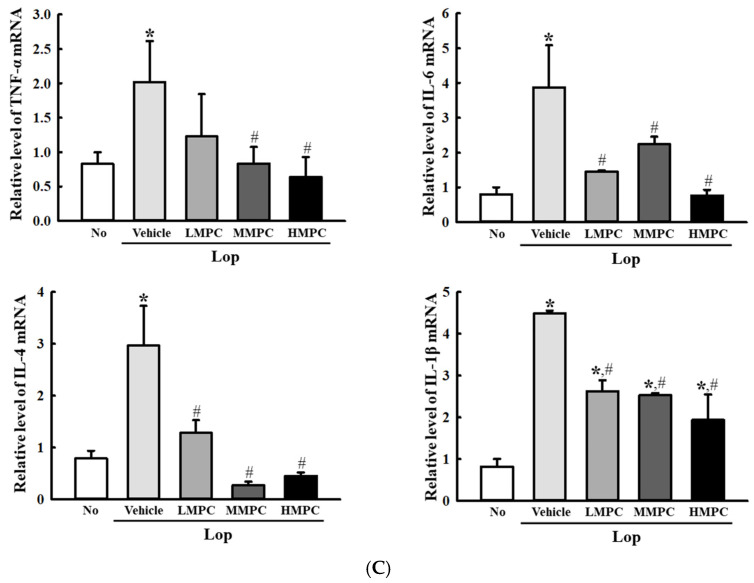
Expression of the TJ key mediators in the mid colon of Lop+MPC-treated SD rats. (**A**) Relative protein levels of E-cadherin and transcription levels of *p120.* The expression level of this protein was measured in the mid colon by Western blot analysis using the specific antibodies and represented as a relative value based on β-actin. (**B**) Transcription level of *claudin-1*, *claudin-4*, *occludin*, and *ZO-1*. (**C**) Transcription level of four inflammatory cytokines, including *TNF-α*, *IL-6*, *IL-4*, and *IL-1β*. The relative level of each gene in the total mRNA of the mid colons was analyzed by RT-qPCR. The data are reported as the mean ± SD. * was represented by a *p* value of less than 0.05 compared to the No group. # was represented by a *p* value of less than 0.05 compared to the Lop+Vehicle-treated group.

Next, we examined whether defecation stimulation effects of MPC are accompanied by alterations in the structure and function of the intestinal epithelial barrier (IEB) in the mid colon of the constipated model. To accomplish this, the expression levels of the key components for tight junction (TJ) and adhere junction (AJ) and inflammatory cytokines were analyzed in the mid colon of the Lop+MPC-treated SD rats. The protein and mRNA levels of the classical cadherin superfamily member (E-cadherin) and the catenin family member (*p120-catenin*) as the core of AJ were remarkably increased in the Lop+MPC-treated group compared to the Lop+Vehicle-treated group (Figure 10A). Also, similar responses were detected in the expression levels of TJ key components, including *occludin*, zonula occludens-1 (*ZO-1*), and *claudin-1*, although their increase rate varied (Figure 10B). Furthermore, the above results were completely reflected in the transcription level of the inflammatory cytokines because the function of inflammatory mediators was characterized in the lamina propria of IEB [38]. The increased mRNA levels of four cytokines, including tumor necrosis factor-α (*TNF-α*), interleukin (*IL*)*-6*, *IL-4*, and *IL-1β*, were remarkably recovered in Lop-induced constipation rats after treatment with MPC (Figure 10C). These results suggest that stimulation effects of MPC in defecation are accompanied by improvement in the structure and function of IEB in the mid colon of the constipated model.

### 3.9. Effects of MPC on the Regulation of GI Hormone in the Tissue of Constipated SD Rats

**Figure 11:**
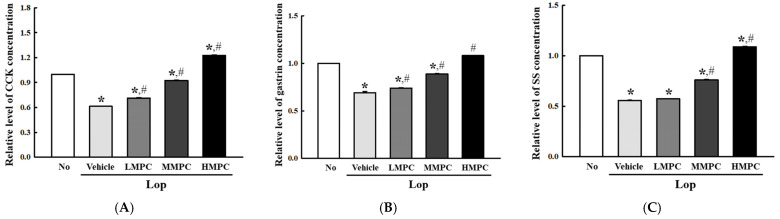
Concentrations of GI hormones in the mid colon of Lop+MPC-treated SD rats. The concentrations of (**A**) CCK, (**B**) gastrin, and (**C**) SS hormone were measured in the mid colon homogenate by an ELISA. They have different minimum detectable concentrations as follows: CCK, 0.1–1000 pg/mL; gastrin, 0.312–20 pg/mL and SS, 1.56–100 pg/mL. The data are represented as the mean ± SD. * was represented by a *p* value of less than 0.05 compared to the No group. # was represented by a *p* value of less than 0.05 compared to the Lop+Vehicle-treated group.

We examined if the stimulation effects of MPC in the Lop-induced defecation delay are accompanied by improvement in the secretion of GI hormone in the constipated model. To achieve this, the cholecystokinin (CCK), gastrin, and somatostatin (SS) concentrations were determined in the mid colon of Lop+MPC-treated SD rats. Three hormones showed similar increase patterns after MPC administration. The CCK, gastrin, and SS concentrations showed a dose-dependent increase in the Lop+MPC-treated groups than in the Lop+Vehicle-treated group, even though the increase rate varied (Figure 11A–C). Above results suggest that the defecation stimulation effects of MPC may be closely related to the regulation of GI hormones in Lop-induced constipation rats.

### 3.10. Verification of Antioxidant Activity of MPC in Lop-Treated pRISMCs

**Figure 12:**
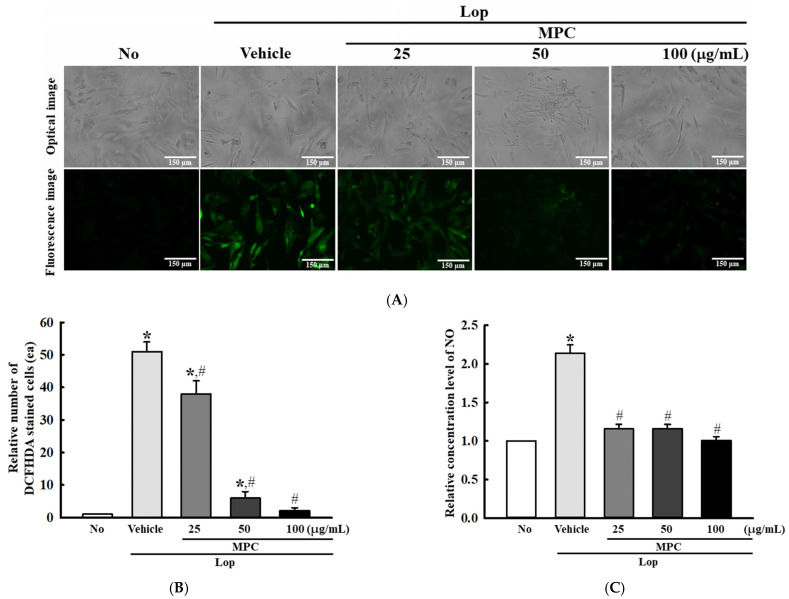
Detection of intracellular ROS in Lop+MPC-treated pRISMCs. (**A**) Images of DCF-stained pRISMCs. The stained cells were detected and counted with a fluorescence microscope at 200× magnification. (**B**) Number of DCF-stained cells. (**C**) NO concentration. The DCF-stained cells were prepared from two to three wells per group, and the fluorescence-positive cells were counted in two positions of a specific view area (67,500 mm^2^) in each well. The NO concentration was measured using Griess reagent. The data are reported as the mean ± SD. * was represented by a *p* value of less than 0.05 compared to the No group. # was represented by a *p* value of less than 0.05 compared to the Lop+Vehicle-treated group.

**Figure 13:**
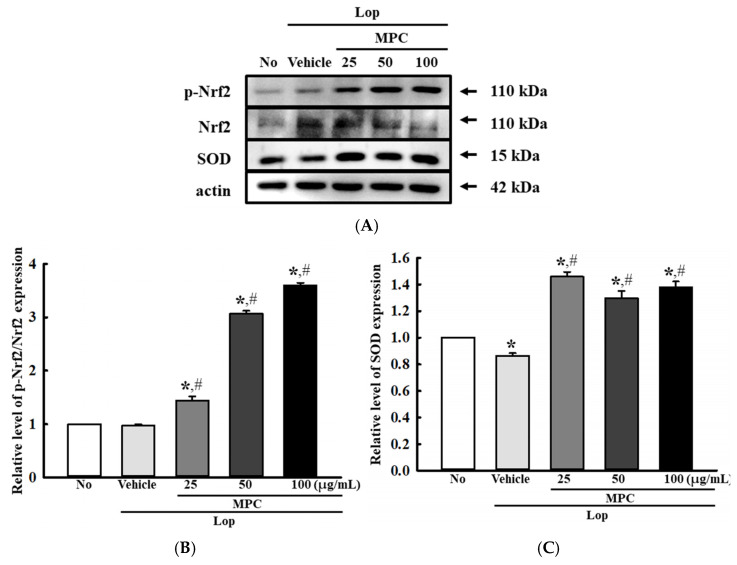
Expression of p-Nrf2/Nrf2 and SOD proteins in Lop+MPC-treated pRISMCs. (**A**) Images for each protein band. (**B**,**C**) Relative levels of each protein. The expression level of each protein was detected in the mid colon using specific antibodies and represented as a relative value based on β-actin. The data are represented as the mean ± SD. * was represented by a *p* value of less than 0.05 compared to the No group. # was represented by a *p* value of less than 0.05 compared to the Lop+Vehicle-treated group.

Furthermore, the antioxidant activity of MPC detected in Lop-induced constipation rats was verified in the Lop+MPC-treated pRISMCs. To achieve this, alterations in the production of intracellular ROS, SOD protein levels, and phosphorylation levels of transcription factors for antioxidant enzymes were analyzed in the Lop+MPC-treated pRISMCs. The levels of Lop-induced ROS and NO production were significantly lower in the pRISMCs after MPC treatment than before (Figure 12A,B). On the other hand, the phosphorylation of Nrf2 and SOD expression were higher in the Lop+MPC-treated group than in the Lop+Vehicle-treated group (Figure 13A,B). These results provide further evidence that the overall results detected in the Lop-induced constipation model may be tightly associated with the recovery of antioxidant defense mechanisms in pRISMCs during Lop-induced constipation.

### 3.11. Verification of Laxative Effects of AbA in Lop-Treated pRISMCs

**Figure 14:**
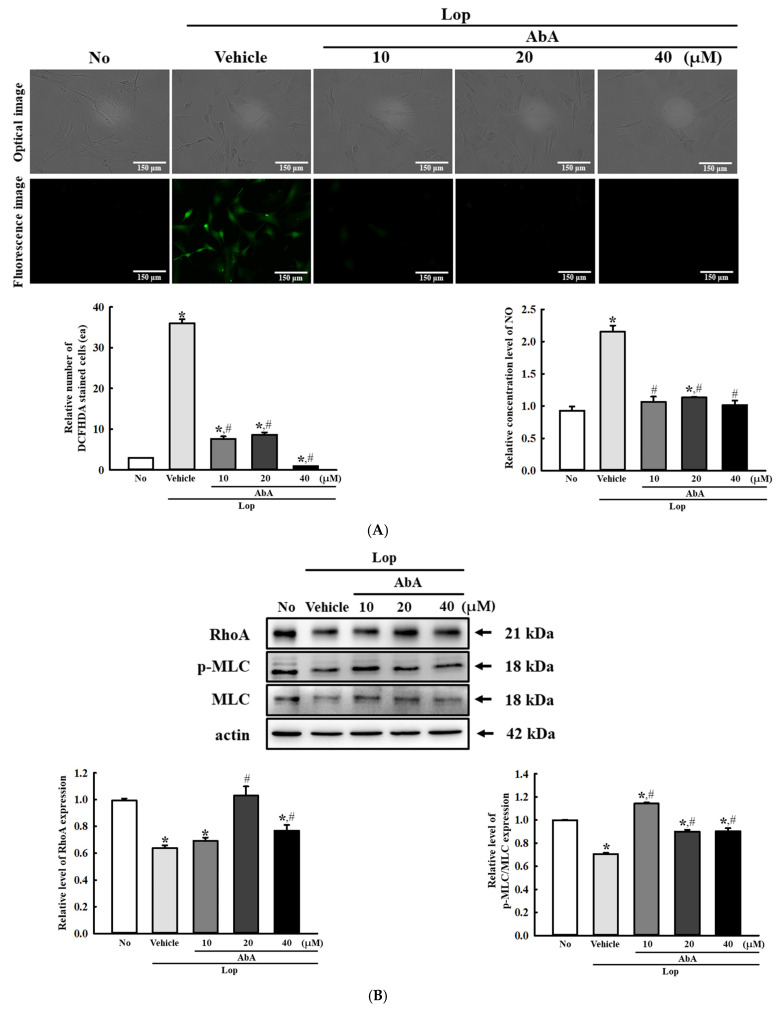
Detection of AbA effects in Lop+AbA-treated pRISMCs. (**A**) Fluorescence image of DCF-stained pRISMCs and NO concentration. The fluorescence of the cells was observed at 200× magnification. The DCF-stained cells were prepared in two wells per group, and the number of fluorescence-stained cells was measured in two positions of specific view area (67,500 mm^2^) in each well. The NO concentration was measured using Griess reagent. (**B**) Expression of RhoA, MLC, and p-MLC proteins in Lop+MPC-treated pRISMCs. The expression level of each protein was detected in the pRISMCs using specific antibodies and represented as a relative value based on β-actin. The data are represented as the mean ± SD. * was represented by a *p* value of less than 0.05 compared to the No group. # was represented by a *p* value of less than 0.05 compared to the Lop+Vehicle-treated group.

Finally, the laxative effects of AbA as a single bioactive compound in MPC were investigated in the Lop+AbA-treated pRISMCs to verify the laxative effects of MPC in the Lop-induced constipation cell model. To achieve this, alterations in the intracellular ROS production, phosphorylation level of myosin light chain (MLC), and expression level of RhoA were analyzed in the Lop+MPC-treated pRISMCs. The increased level of intracellular ROS and NO production in the Lop+Vehicle-treated group was decreased with treatment of AbA (Figure 14A). During this antioxidant activity, the phosphorylation level of MLC and expression level of RhoA were remarkably recovered in the Lop+AbA-treated group, although it was not a dose-dependent change (Figure 14B). These results suggest that the laxative effects of MPC are strongly linked to AbA, the main ingredient of MPC.

## 4. Discussion

The improvement effect against constipation has been investigated in various products, including some nutraceuticals, probiotics, and several laxatives, including stimulant or osmotic laxatives, prosecretory drugs, and 5-hydroxytryptamine 4 (5-HT4) agonists, as well as botanical laxatives, including senna, cascara, frangula, aloe, and rhubarb [39]. Nevertheless, these strategies do not provide sufficient patient satisfaction because of the high refractory, low efficacy, and various side effects [40]. Among them, natural products have been considered beneficial treatment strategies for constipation based on their low toxicity and side effects, easy acquisition of materials, and efficiency for disease control and prevention [39,41]. As part of this research, this study evaluated the antioxidant activities and laxative effects of MPC in Lop-induced constipation SD rats to identify novel laxatives based on their antioxidant activity. The results of the present study provide significant evidence that MPC with high antioxidant activity have great potential for constipation treatments, but more research is needed to support their pharmacological action.

The possibility of natural products based on antioxidant activity as medicines for constipation was verified in Lop-induced constipation animal models. Thus far, four natural products, including *Malva sylvestris*, *Boehmeria nivea* L., *Ecklonia cava*, and *Cistanche deserticola*, and one synthetic product, T–X, have laxative effects accompanied by antioxidant capabilities [17,18,19,20,21]. Most of them induce an increase in the expression and activity of antioxidant enzymes, including glutathione peroxidase (GSH-Px), SOD, and catalase (CAT), as well as inhibiting lipid peroxidation and hydrogen peroxide production during the improvement in constipation symptoms [17,18,19,20,21]. In particular, *Ecklonia cava* and T–X exhibited high scavenging activity against DPPH and ABTS radicals, even though there were some differences in IC_50_ values [19,21]. This study investigated the antioxidant activities of MPC in a Lop-induced constipation animal model and primary intestinal muscle cells. MPC inhibited NADPH oxidase transcription and enhanced antioxidant enzyme expression in Lop-treated SD rats and pRISMCs. These results were very similar to the results of previous studies that investigated the possibility of natural products based on antioxidant activity as medicines for constipation, even though antioxidant activity analysis has been performed in various tissues [17,18,19,20,21].

The bioactive components in natural products with antioxidant activity and laxative effects are essential for understanding the mechanism of action for improving constipation. Generally, laxatives have been classified into four groups, including bulking agents, osmotic agents, stimulating agents, and lubricating agents, based on their action mechanisms [42]. These groups include various substances, such as psyllium, methylcellulose, saline, mannitol, senna, cascara, liquid paraffin, and arachis oil derived from natural products [42]. In particular, several phytochemicals, including saponins, flavonoids, tannins, sterols, terpenoids, alkaloids, phenolic compounds, and spicatoside A, were detected in natural products with laxative effects [43,44,45]. Most of them play a role as antioxidants that neutralize free radicals and reduce their power to cause cellular damage [46]. Four natural products with laxative effects accompanied by antioxidant capabilities contain many bioactive compounds. *Ecklonia cava* contains phlorotannins, fucoidans, protein derivatives, and carotenoids, while many phenolic compounds and flavonoids responsible for its strong antioxidant activity were detected in *Malva sylvestris* [47,48]. In addition, *Boehmeria nivea* showed high levels of linoleic acid, linolenic acid, total phenolic, and flavonoids [49]. Furthermore, diverse chemical constituents, including benzyl alcohol glycosides, iridoid terpenes, lignans, phenylethanol glycosides, polysaccharides, and other compounds responsible for significant pharmacological effects, were identified in *Cistanche deserticola* [50,51]. In this study, we assessed the laxative effect and antioxidant activity of *Pinus densiflora* extract. This product contained several bioactive compounds such as dehydroabietic acid, 12-hydroxyabietic acid, ferruginol, abietic acid, taxodone, and 15-hydroxydehydroabietic acid. Nevertheless, these compound detections in MPC were different from previous studies that analyzed the biological properties and functional compounds in butanol extracts from green pine cones of *Pinus densiflora* [27,52,53]. The studies using butanol extracts detected only a few compounds, including digalloyl hexose, caffeoyl-O-hexo-galloyl moiety, and procyanidins. Furthermore, comparisons with previous studies on the distribution of bioactive compounds in *Pinus densiflora* are limited. This is because there are very few papers on this information thus far, and most reported papers have focused on their needles and roots [54,55,56]. Therefore, our findings on green pine cones are valuable because they provide novel information on bioactive compounds in MPC.

The abilities of natural products to improve constipation have been determined by various indicators, including stool parameters, GI transit, histological structure of the mid colon, GI hormone secretion, and mucin production [57]. In general, three medicinal herbs, rhubarb, senna, and aloe, are commonly used to treat constipation, even though the countries differ [58]. Senna, fruit, and leaves of *Senna alexandrina* improve constipation by stimulating bowel movement and peristalsis and enhancing the water contents of stools [59,60]. Also, very similar effects on the mitigation of constipation were observed in the root and rhizome of rhubarb [61,62]. Their extracts stimulate mucus production, improve GI motility, and change the intestinal flora [63,64]. Aloe or *Aloe vera* has the potential to relieve constipation because its administration improves intestinal motility, defecation, and abnormal changes in body weight in Lop-induced constipation models [65,66]. Furthermore, significant laxative effects were investigated in constipation models after treatments with other plant products, including *Moringa oleifera Lam.*, *Allium mongolicum Regel*, *Chrozophora tinctorial*, *Globularia alypum*, *Liriope platyphylla*, and *Dendropanax morbiferus* [64,67,68,69,70,71,72]. In this study, several key parameters, including stool excretion, GI motility, colon length, histological structure of the colon, mucin secretion, and GI hormone concentration, were analyzed in Lop-induced constipation SD rats after an MPC treatment to verify the laxative effects of MPC. Most of the indicators analyzed in this study changed in a similar manner to those of previous studies. In addition, they presented scientific evidence that MPC can be considered a laxative for constipation. Nevertheless, further studies will be needed to establish a clear correlation between mechanisms for the laxative effects and bioactive components contained in MPC.

Furthermore, the histological structure of the colon includes the mucosa, submucosa, muscularis, and serosa/adventitia layer [73]. Each layer has its own antioxidant system. The mucosa layer has the most abundant antioxidant system, including a high level of antioxidant enzymes and a non-enzymatic free-radical scavenger, glutathione (GSH) [74]. The mucus produced by the goblet cells protects the mucosa layer by blocking direct contact between ROS and toxins [75]. Also, the submucosa layer is protected by similar enzymes found in the mucosal layer [76,77]. However, in the muscularis layer, ROS were majorly removed with Mn-SOD in the matrix of mitochondria [78]. During constipation, the production of ROS was significantly increased, and the activity and expression levels of SOD and catalase were decreased [79,80]. Therefore, the treatment of antioxidants significantly recovered the tissue structure of the colon, although no scientific evidence has been provided for the molecular mechanisms by which these changes are clearly reflected in the changes of histological structure in the colon. The tannin-enriched extract of *Ecklonia cava* with high antioxidant activity induces the structural recoveries of the thickness of the flat luminal surface and mucosa, the crypts of Lieberkuhn, and the shape of goblet cells [19]. Also, similar restorations were detected in the Lop-induced constipation model after treatment with *Cistanche deserticola* crude polysaccharides (CDCP) and ramie (*Boehmeria nivea* L.) leaf extract as natural antioxidants [18,20]. In this study, we used MPC as antioxidants to improve the constipation in the Lop-induced model. As in previous studies, structural improvements in the colon have similarly been observed in the Lop+MPC-treated model. All the above results suggest that antioxidants are associated with alterations in the histological parameters of the colon, although further molecular biological studies are needed.

Meanwhile, three junctional complexes, including AJ, desmosomes, and TJ, play an important role as the key selective physical barriers between the intestinal lumen and internal tissue in the GI tract [81,82]. Among them, TJ plays a major role in sealing between the adjacent epithelial cells and consists of transmembrane proteins (claudin, occluding, and tricellulin) and cytoplasmic plaque proteins (zona occludens) [82,83,84,85]. Alterations in the major components of TJ were detected in the constipation model animals after treatment with a few natural products, although there is a difference in the rate of change. The expression levels of three TJ components, including *occludin*, *ZO-1*, and *claudin-1*, except *claudin-4*, were significantly recovered in the Lop-induced constipation rat model after treatment with phlorotannin (Pt) [86]. Also, other changes, such as increased *ZO-1*, decreased *claudin-1*, and no change in *occludin*, were detected in Lop-induced constipation mice after treatment with Wenyang Yiqi Decoction (WYD) [87]. Furthermore, AJ-containing transmembrane proteins (E-cadherin and nectins) and intracellular components (p120-catenin, β-catenin, and α-catenin) as their main components play an important role in initiating cell–cell contact [38,88,89,90]. The decreased levels of E-cadherin and p120 in Lop-induced constipation were remarkably increased after treatment of Pt [86]. In the present study, we investigated the expression levels of two AJ and four TJ compounds in the colon of Lop+MPC-treated rats. Significant enhancement in their mRNA level was detected after MPC treatment, although the increase rate varied. These findings are similar to those of previous studies that analyzed the Lop+Pt-treated model. But many differences in the expression level of TJ components were identified between our study and Lop+WYD-treated mice. We thought that this difference between them can be attributed to diversity in the pathological severity caused by animal species and bioactive component distribution in treatment products.

In addition, the GI hormones, including CCK, gastrin, and SS, play an important role in the regulation of ENS activities to control the complex digestive process [91]. During constipation, the concentrations of these hormones were significantly decreased in the colon of Lop-induced constipation animals and human patients [19,92]. However, these alterations in the concentration of GI hormone were remarkably recovered by natural products with laxative effects [19,93]. In the present study, we analyzed the concentrations of three GI hormones in Lop+MPC-treated rats. A dose-dependent pattern in the increase in GI hormones was detected in the Lop-induced constipation model after treatment with MPC. The results of the present study were very consistent with those of previous studies.

Lastly, the laxative effects of MPC on Lop-induced constipation were compared to those of bisacodyl [4,4′-diacetoxy-diphenyl-(pyridyl-2)-methane] as another laxative drug. This drug is a diphenylmethane derivative stimulant laxative to consider the improvement in bowel function, constipation-related symptoms, and disease-related quality of life [94]. Bisacodyl can mediate dual functions: an anti-absorptive-secretory effect as well as a direct prokinetic effect in the colon [95]. As shown in Supplementary Table S3, five parameters for laxative effects were compared between the MPC-treated group and the bisacodyl-treated group reported in a previous study to estimate the potential of MPC as a candidate for a medicine [96]. Among them, five parameters, including stool number, stool weight, mucosal layer thickness, muscle layer thickness, and PI3K phosphorylation, were significantly improved in the MPC-treated group than in the bisacodyl-treated group. However, the other two parameters, including stool water contents and mAChRs M2 expression levels, were higher in the bisacodyl-treated group than in the MPC-treated group. Therefore, these comparison results show that MPC has great potential for laxative drugs like bisacodyl, although few differences are expected in the mechanism of their action.

## 5. Conclusions

This study investigated the antioxidant activities and laxative effects of MPC in Lop-induced constipation rats and pRISMCs as part of a study to find a new medicine for constipation. These results show that MPC had high antioxidant potential and activated the antioxidant defense mechanism in the mid colon of Lop-induced constipation rats. In addition, these results provide novel evidence that MPC treatment can improve many critical parameters for constipation in the same model as laxative effects. These effects were also detected in the Lop-treated pRISMCs after MPC treatment. Overall, these results suggest that MPC is a novel therapeutic product for constipation associated with oxidative stress. Nevertheless, this study had some limitations. The present study did not verify the mechanism of action of which compounds were directly associated with the therapeutic efficacies because the extract contains too many compounds. Therefore, further studies will be needed to determine if administering a single bioactive compound with high antioxidant activity can be considered as another treatment option to improve the chronic constipation caused by oxidative stress in an animal model.

## Data Availability

Data is contained within the article and Supplementary Materials.

## Supplementary Materials

The following supporting information can be downloaded at: https://www.mdpi.com/article/10.3390/antiox14010037/s1, Figure S1: Preliminary experimental results for determining MPC concentration; Figure S2: Experimental scheme for the MPC treatment in (A) Lop-induced constipation SD rats and (B) Lop-treated pRISMCs; Figure S3: Chemical structures of 10 identified compounds. Table S1: RT-qPCR primer list of target genes; Table S2: Antibodies list for western blot analyses; Table S3: Comparison of MPC and bisacodyl effects in Lop-induced constipation.
