# Supplementary material for: Antioxidant and Laxative Effects of Methanol Extracts of Green Pine Cones (Pinus densiflora) in Sprague-Dawley Rats with Loperamide-Induced Constipation
Source: Antioxidants (Basel). 2024 Dec 31;14(1):37. doi: 10.3390/antiox14010037 (PMC11762744; doi:10.3390/antiox14010037)
Supplement: Supplementary file 1 [file antioxidants-14-00037-s001.zip › Supplementary Figure.pdf]

Supplementary Figures

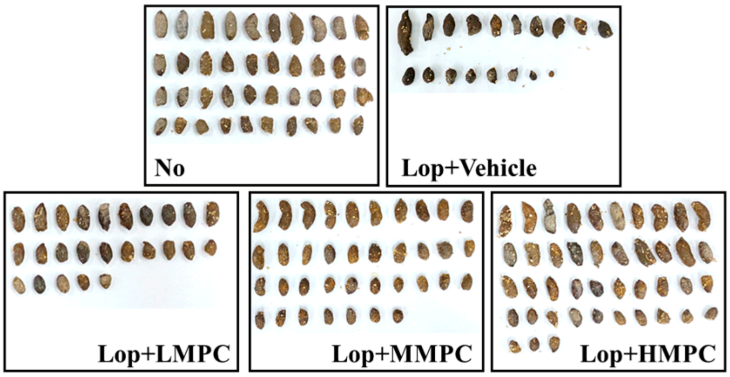

(A)

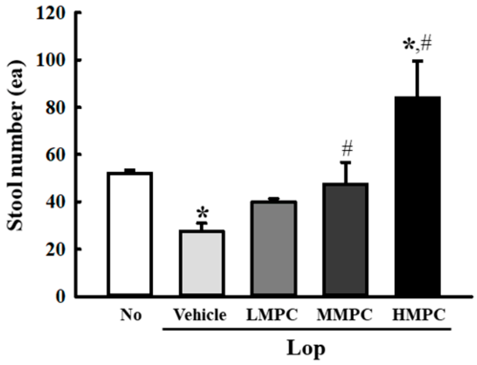

(B)

Supplement Figure S1. Preliminary experimental results for determining MPC concentration. (A) Stool morphology and (B) Stool number. * was represented a *p* value of less than 0.05 compared to the No group. # was represented a *p* value of less than 0.05 compared to the Lop+Vehicle-treated group.

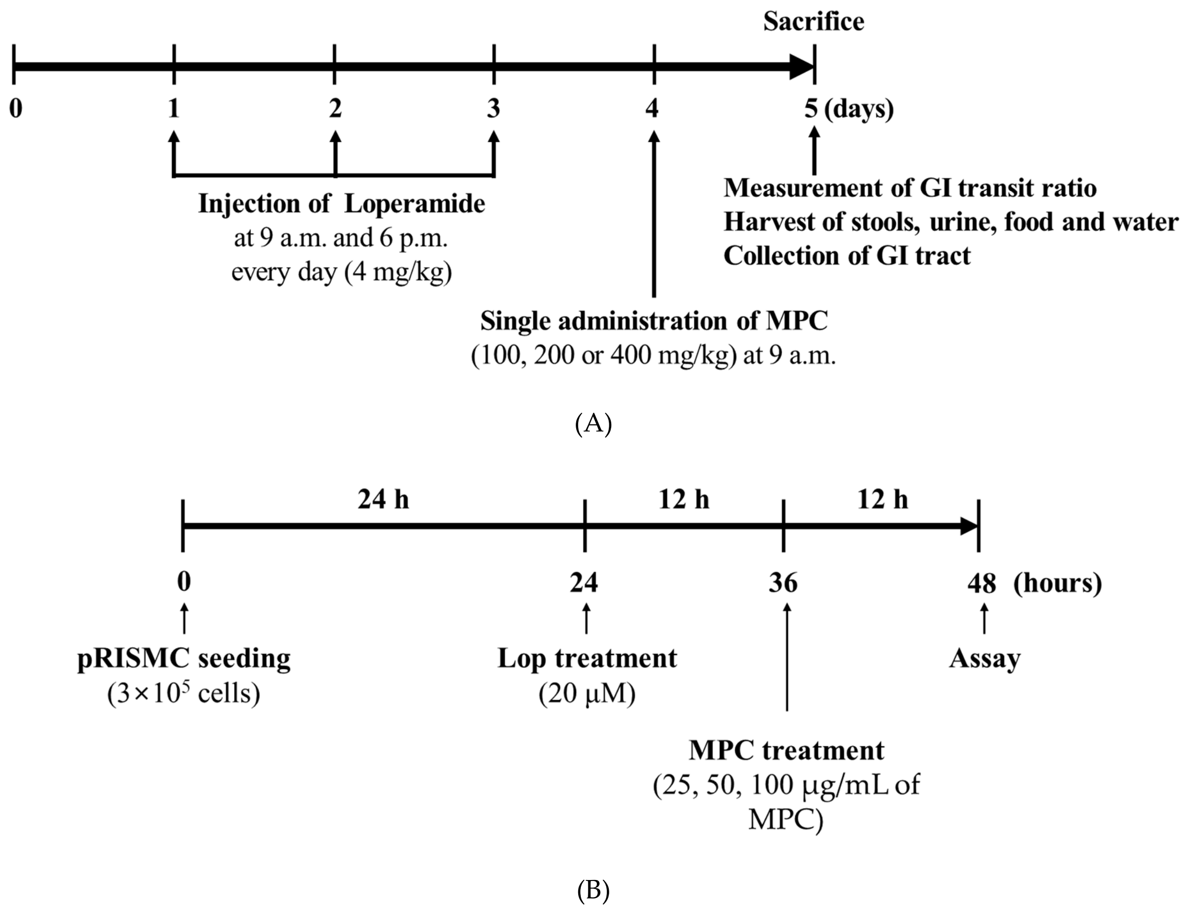

Supplement Figure S2. Experimental scheme for the MPC treatment in (A) Lop-induced constipation SD rats and (B) Lop-treated pRISMCs.

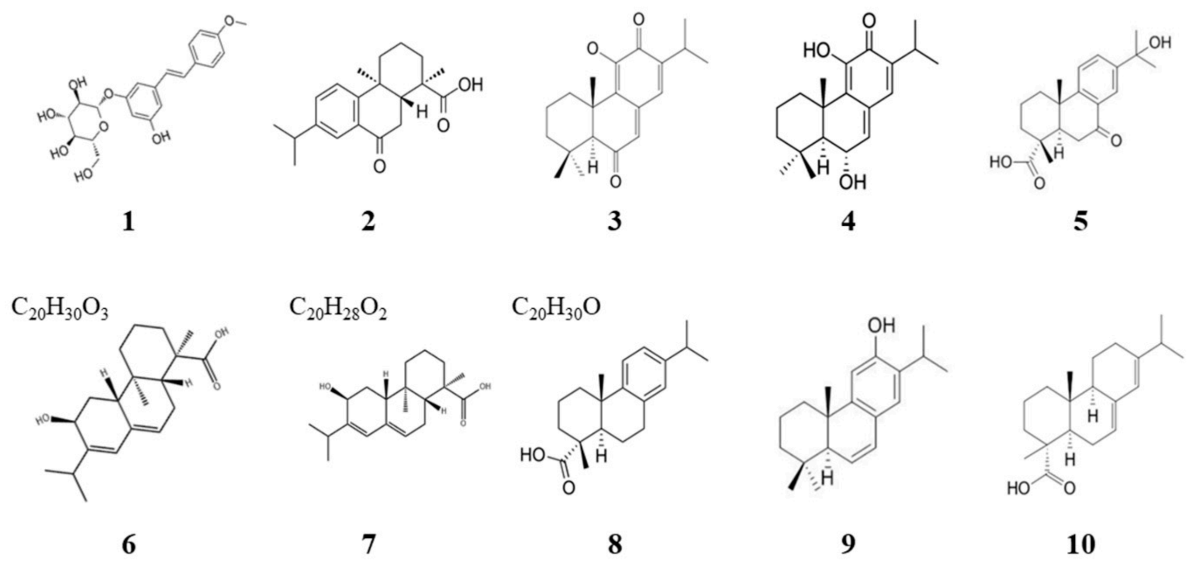

Supplement Figure S3. Chemical structures of 10 identified compounds. 1: Desoxyrhaponticin; 2: Oxodehydroabietic acid; 3: Taxodione; 4: Taxodone; 5: 15-hydroxydehydroabietic acid; 6: 12-hydroxyabietic acid; 7: Dehydroabietic acid; 8: Ferruginol; 9: Dehydroferruginol; 10: Abietic acid.
