# Supplementary material for: Antioxidant and Laxative Effects of Methanol Extracts of Green Pine Cones (Pinus densiflora) in Sprague-Dawley Rats with Loperamide-Induced Constipation
Source: Antioxidants (Basel). 2024 Dec 31;14(1):37. doi: 10.3390/antiox14010037 (PMC11762744; doi:10.3390/antiox14010037)
Supplement: Supplementary file 1 [file antioxidants-14-00037-s001.zip › Supplementary Table.pdf]

Supplementary Table S1. RT-qPCR primer list of target genes

**Gene name**	**Sequence (from 5' to 3')**
MUC2	
Forward	GCA CAT TCC TTC GCA TCT TAA A
Reverse	AAA GCA AAG AAT GGA ACA GAA CAG AAA CTC
AQP3	
Forward	GGTGG TCCTG GTCAT TGGAA
Reverse	AGTCA CGGGC AGGGT TGA
AQP8	
Forward	TCGCT GGCAG TCACA GTGA
Reverse	TCCAA ATAGC TGGGA GATCC A
NOX4	
Forward	AGCAT CTGCA TCTGT CCTGA AC
Reverse	ACTGT CCGGC ACATA GGTAA AAG
NOX1	
Forward	CCCTC GGACT TTGGC AAA
Reverse	CCAGA CTCGA GTATC GCTGA CA
DUOX2	
Forward	GGCGC TGGAA GCCTC TTAC
Reverse	AAAAG GGTTT TTGCG GGTAG A
Claudin-1	
Forward	CCCCG GAAAA CAACC TCTTA C
Reverse	TGTCA CACAT AGTCT TTCCC ACTAG AA
Claudin-4	
Forward	CGTGG CAAGC ATGCT GATTA
Reverse	GTCGC GGATG ACGTT GTG
ZO-1	
Forward	CCTCC GTTGC CCTCA CAGTA
Reverse	GGGCG CCCTT GGAAT G
Occludin	
Forward	TTGAA GAGTG GGTTA AAAAT GTGTC T
Reverse	TCAAC TCTTT CCGCA TAGTC AGAT
TNF-α	
Forward	ATCCG CGACG TGGAA CTG
Reverse	ACCGC CTGGA GTTCT GGAA
IL-6	
Forward	TTCCA TCCAG TTGCC TTCTT G
Reverse	GGGAG TGGTA TCCTC TGTGA AGTC
IL-4	
Forward	CGCCA TGCAC GGAGA TG
Reverse	CGAGC TCACT CTCTG TGGTG TT
IL-1β	
Forward	CTACA GGCTC CGAGA TGAAC AAC
Reverse	TCCAT TGAGG TGGAG AGCTT TC
p120	
Forward	TGGAC CATGC GCTAC ACGCC
Reverse	CCGAA GTTTC CGCCG GGCTT
β-actin	
Forward	TGGAA TCCTG TGGCA TCCAT GAAAC
Reverse	TAAAA CGCAG CTCAG TAACA GTCCG

Supplementary Table S2. Antibodies list for western blot analyses

Antibody	Company	Catalog number
Anti-Nrf2	Abcam Com., Cambridge, UK	Ab137550
Anti-p-Nrf2	Invitrogen Co., Ltd, Carlsbad, CA, USA	PA5-67520
Anti-SOD	Abcam Com., Cambridge, UK	Ab13498
Anti-E-cadherin	Cell Signaling Technology Inc., Cambridge, MA, USA	#3195
Anti-RhoA	Cell Signaling Technology Inc., Cambridge, MA, USA	#2117
Anti-MLC	Abcam Com., Cambridge, UK	Ab92721
Anti-p-MLC	Cell Signaling Technology Inc., Cambridge, MA, USA	#3671
Anti-β-actin	Cell Signaling Technology Inc., Cambridge, MA, USA	#4967

Supplementary Table S3. Comparison of MPC and bisacodyl effects in Lop-induced constipation

**Categories**	**Alteration rate after treatment of each drug**			
	**LMPC**	**MMPC**	**HMPC**	**Bisacodyl**
Stool number (ea)	148.1±12.1	173.6±6.3	318.8±12.2	71.1±2
Stool weight (g)	174.7±31.1	173.2±2.2	148.8±33.4	169.1±7.5
Stool water contents (%)	50.45±4.3	49±0.4	54±4.8	149.1±5.7
Mucous layer thickness (μm)	54.9±1.3	85±0.5	93.6±3.8	213.7±5.5
Muscle layer thickness (μm)	72.5±22.2	119.7±27.5	179.1±30.8	37±7.5
